# Assessing Evidence-Based Practice in Operating Room Nursing Students: A Cross-Sectional Study in the Southwest of Iran

**DOI:** 10.1155/2024/5552711

**Published:** 2024-05-09

**Authors:** Jamshid Eslami, Amir Arsalan Saeedi, Majid Najafi Kalyani

**Affiliations:** ^1^Department of Anesthesia, School of Nursing and Midwifery, Shiraz University of Medical Sciences, Shiraz, Iran; ^2^Student Research Committee, School of Nursing and Midwifery, Shiraz University of Medical Sciences, Shiraz, Iran; ^3^Department of Medical Emergencies, School of Nursing and Midwifery, Shiraz University of Medical Sciences, Shiraz, Iran

## Abstract

Evidence-based practice (EBP) is a clinical decision-making process that is grounded in the utilization of the most reliable and up-to-date evidence. It involves utilizing resources and evidence to enhance patient management. The application of evidence-based decisions in patient care and education is of utmost importance in the performance of health sciences students. However, the emphasis on this concept among operating room nursing students has been lacking. Hence, the objective of this research is to assess the implementation of evidence-based practice among operating room nursing students studying in the operating room department at the Shiraz University of Medical Sciences in Iran. The study follows a descriptive, cross-sectional design, with a sample of 148 operating room nursing students selected through census sampling based on the student list. After obtaining informed consent, participants completed a demographic information form and the Rubin–Parrish evidence-based practice questionnaire. The data that were gathered underwent analysis through the utilization of SPSS version 22 software, employing descriptive statistics, *T*-tests, and Pearson correlation coefficient tests. The results revealed that the overall average score of evidence-based practice among the students exceeded the standard scores (172.66 ± 14.74). There was a significant association between the evidence-based practice score and prior familiarity with evidence-based practice, interest in the field of study, research experience, intention to participate in the Master's exam, and the participants' grade point average (GPA) (*p* < 0.05). These findings indicate that operating room nursing students displayed an average level of evidence-based practice, emphasizing the need for effective plans and strategies to improve their performance. Addressing the identified factors from this study becomes crucial in this regard.

## 1. Introduction

Evidence-based medicine is a contemporary approach within the medical field that integrates clinical experiences and the findings of experimental studies. By doing so, it provides the best available evidence to inform accurate and informed diagnostic and clinical decision-making [1]. Research suggests that familiarity with the fundamentals of EBP among medical science students can enhance their clinical knowledge and skills and lead to a shift in behavior and perspective [2]. Moreover, it is widely believed that the engagement of medical science students in evidence-based educational programs fosters the enhancement of their critical thinking skills [3].

The significance of research and evidence-based practice in the field of operating room nursing students to enhance the standard of patient care is underscored by the Association of Formal Surgical Nurses. According to this association, studies should serve as the basis for practice in operating rooms. Evidence-based performance contributes to enhancing the quality of patient care, and utilizing research findings in the practice of operating room nursing students is essential for achieving better patient outcomes [4].

Despite the growing emphasis on evidence-based practice in the medical sciences over the past decades, it is still evident that many healthcare decisions continue to be influenced by traditional methods, assumptions, personal experiences, and individual opinions and skills. It is essential to possess adequate knowledge and competence in gathering and evaluating evidence and implementing best practices within a clinical setting [5]. Multiple research studies have provided evidence that a considerable portion of healthcare practitioners lack the essential expertise and competencies needed to effectively execute evidence-based practice [6]. Support plays a vital role in assisting individuals in acquiring new knowledge and skills [7]. While evidence-based healthcare is considered fundamental for operating room nurses, its practical implementation can present challenges. The understanding that operating room nurses possess regarding this process can significantly impact their performance [8]. Although the basics of EBP are included in the educational curriculum of many medical science faculties worldwide, there is a paucity of studies addressing the motivating factors and challenges associated with its education [9]. The study's findings revealed that evidence-based education has the potential to augment the knowledge, attitudes, and skills of undergraduate nursing students. Nevertheless, the available evidence is inadequate to substantiate the assertion that evidence-based education directly translates into enhancements in students' behaviors [10].

The education of medical sciences students has been greatly affected by the emergence of the COVID-19 pandemic, leading to significant impacts on various aspects of the healthcare system [11, 12]. Consequently, given the importance of evidence-based practice and the lack of sufficient and appropriate literature in the field of Operating Room majors, we aim to contribute to the development of plans, policies, and educational interventions by evaluating the practice and acceptance of operating room students towards evidence-based practice and its related factors. The results of this study can potentially enhance the adoption of evidence-based practice and improve the quality of patient care through the training of competent operating room nurses who can effectively utilize both evidence-based practice and clinical problem-solving approaches. Moreover, it is expected that such an approach will contribute to a reduction in surgical errors. In light of the aforementioned research gap and concerns, the present study aims to assess evidence-based practice among operation room students at the Shiraz University of Medical Sciences in the year 2021.

## 2. Materials and Methods

### 2.1. Study Design

This study utilized a cross-sectional research design. The study included a sample of 148 operating room nursing students who were studying at the Shiraz University of Medical Science in the year 2021.

### 2.2. Sampling

The sampling method used in this study was a census approach, based on the list of all the students enrolled in the 6th, 7th, and 8th semesters of the operating room program in schools affiliated with the Shiraz University of Medical Sciences. The study encompassed a cohort of 148 individuals who were extended invitations to partake in the research.

Initially, informed consent was acquired from all participants. Then, a link to the online questionnaire, which consisted of the demographic information form and the desired questionnaire, was sent to the students through social networks such as WhatsApp. To ensure complete responses, the online questionnaire was designed in a way that required participants to answer all questions before submission. Once the sampling process was completed, the data collected underwent analysis through the utilization of SPSS software version 22.

### 2.3. Measurements

The data collection tools in this study consisted of two questionnaires.

Demographic characteristics form: This questionnaire included two parts. One part aimed to gather educational information (e.g., GPA, interest in the field of study, study semester, research experience, etc.), while the other part focused on personal information such as age, gender, and marital status.

Rubin and Parrish's (2010) evidence-based practice questionnaire: Rubin and Parrish (2010) originally developed and validated this questionnaire in the United States of America [13]. It comprised 51 statements categorized into five areas: knowledge, attitude, possibility of implementing evidence-based practice, willingness to implement evidence-based practice, and recent usage of evidence-based practice. The knowledge and willingness sections included 10 statements each, the attitude section contained 14 statements, and the possibility and recent usage sections comprised 7 and 10 statements, respectively. The survey employed a 5-point Likert scale, which spanned from “strongly disagree” (score 1) to “strongly agree” (score 5). Higher scores indicated a stronger alignment with evidence-based practice. The validity of the questionnaire was assessed by Ashktorab et al. in Iran, with experts evaluating its face and content validity (0.98). The scale's reliability was further validated, resulting in a Cronbach's alpha coefficient of 0.89 [14].

### 2.4. Ethical Consideration

The present study has obtained approval and registration from the Research Ethics Committee of the Shiraz University of Medical Sciences, with the code IR.SUMS.REC.1400.076. Adequate assurance was provided to all participating students regarding the confidentiality of their information. It was explicitly communicated to them that they possessed the right to withdraw from the study at any point if they chose not to proceed.

### 2.5. Data Analysis

The data were analyzed using descriptive statistics such as frequency, percentage, mean, and standard deviation. To evaluate the normality of the data distribution, the Kolmogorov–Smirnov test was utilized. Furthermore, the relationships between variables were examined using the Pearson correlation coefficient test, while the *T*-test was employed to assess the differences between variables. A significance level of *p* < 0.05 was deemed to be statistically significant.

## 3. Results

In this study, a total of 148 students participated. Among the participants, 52.7% were female and the majority of them (91.2%) were single. In terms of educational background, 49.3% of the participants were in their sixth semester. Regarding research experience, the majority of participants (71.6%) did not have any previous research experience and a significant proportion of them (78.4%) were not familiar with evidence-based practice. The average grade point average (GPA) of the participants in the previous semester was 16.73 ± 1.01.  Table 1 presents the overall score of the participants on the evidence-based practice (EBP) scale, which was found to be 172.66 ± 14.74.

Table 2 shows the results of the *T*-test analysis. The findings indicate that there was no significant difference between gender and the average score of evidence-based practice (*p*=0.740). However, significant differences were observed in relation to marital status (*p*=0.039), interest in the field of study (*p*=0.001), willingness to participate in the Master's entrance exam (*p*=0.019), participation in the evidence-based practice course (*p*=0.003), and research experience (*p*=0.001).

Furthermore, the results of the Pearson correlation coefficient test, as shown in Table 3, revealed a positive and significant relationship between GPA and the average score of evidence-based practice (*p*=0.001). However, the correlation between age and the evidence-based practice average score was not statistically significant (*p*=0.367).

## 4. Discussion

According to the study results, the overall score of the participants on the evidence-based practice scale was above average, specifically 172.66 ± 14.74. Additionally, all the students obtained above-average scores in all subscale aspects, with the interest factor having the highest average score and the attitude factor having the lowest average score. These findings are consistent with a study conducted in China by Hong and Chen, which also reported that most doctors had an average level of evidence-based practice [15].

Regarding the demographic factors, no significant differences were found between gender, passing the research method course, and age of the participants in relation to EBP scores. However, a study conducted among undergraduate nursing students in Colombia, Chile, and Spain [16] showed a significant difference between the length and passing of the research method course and evidence-based practice, which contrasts with the present study's findings. This discrepancy may be attributed to variations in the quality and content of the course, as a poorly presented course may have minimal impact on students' practices.

Moreover, the investigation unveiled notable statistical variances between the scores of evidence-based practice and the marital status as well as the previous acquaintance with evidence-based practice among the participants, interest in the field of study, research experience, willingness to participate in the Master's entrance exam, and student GPA. Single participants, those familiar with evidence-based practice, those interested in their field of study, participants with research experience, those willing to pursue a Master's degree, and students with higher GPAs obtained higher scores on the evidence-based practice scale. These findings align with a study conducted by Khodadadi et al. which demonstrated a significant relationship between knowledge and attitude towards evidence-based practice and factors such as Internet access, use of reference books, participation in evidence-based practice classes, and engagement in research work. Emphasizing the importance of evidence-based practice, it is crucial to implement proper planning and educational reforms in the medical student curriculum to foster the development and promotion of evidence-based practice [17].

The findings of the present study are consistent with several previous studies. Ruzafa-Martinez et al. demonstrated that students who participated in evidence-based practice classes had higher evidence-based practice qualification scores, supporting the results of the present study [18]. Similarly, studies conducted by Kebbi et al. and Rojana et al. showed that students who underwent evidence-based practice educational programs experienced significant improvements in evidence-based practice [19, 20]. Additionally, Mena-Tudela et al. reported that educational interventions focused on evidence-based practices and emergency incident techniques enhanced evidence-based practice qualification in second-year students during their internships [21]. These studies align with the findings of the present study, emphasizing the importance of implementing educational interventions and practical classes to enhance evidence-based practice.

Research conducted by Rebecca et al. highlighted a direct relationship between research activities and clinical and professional performance [22]. Students engaged in research activities demonstrated higher competency in using evidence to deliver patient care, as research provides a deeper understanding of evidence-based practice.

In a recent investigation conducted by Amit-Aharan et al., it was discovered that nursing students who possess a favorable outlook towards evidence-based practice exhibit a noteworthy and constructive correlation with their internal academic motivation. This, in turn, contributes to a heightened likelihood of implementing evidence-based practice in their future endeavors [23]. This study's results are consistent with the present study, which reported a significant relationship between students' GPA, interest in the field of study, and motivation to continue studying with evidence-based practice scores. Higher academic motivation is indicative of the integration of evidence and scientific knowledge in clinical practices. These findings can inform effective planning and improvement of evidence-based practice in operating room students by healthcare experts and educational policymakers.

The incorporation of evidence-based practice into educational programs and curricula plays a vital role in molding the professional knowledge, skills, and attitudes of students, ultimately improving the standard of care delivered. Comprehensive incorporation of evidence-based practice into clinical curricula and its inclusion as a key competency in professional standards and requirements can elevate evidence-based performance in students [24, 25].

Given the obstacles presented by the COVID-19 pandemic [26], it is crucial to recognize its influence on the education of students pursuing medical sciences and the delivery of evidence-based practice [27]. Educators should assess evidence-based practice competence in clinical learning settings and strive to improve the knowledge and skills of their students.

A significant strength of this study is that it is the first of its kind in Iran, focusing on operating room students. However, one potential limitation is the concurrent COVID-19 pandemic, which restricted the researchers' access to study participants. Consequently, online questionnaires were utilized, and although efforts were made to ensure accurate responses, there is a possibility of some participants providing inaccurate answers, which could have influenced the study results.

## 5. Conclusion

To summarize, it is of utmost importance to equip undergraduate students with sufficient proficiency in evidence-based practice (EBP) in order to fulfill the requirements of their future professional endeavors. By systematically incorporating EBP knowledge and skills into the formal curriculum, students' learning experiences and outcomes can be greatly enhanced. Educational planners and policymakers should devise effective plans and strategies to enhance EBP. The factors identified in this research should be duly acknowledged and addressed to facilitate the successful implementation of EBP.

## Figures and Tables

**Table 1:** The average score of the evidence-based practice scale and its subscales in the participants.

Variable	Mean ± SD
Knowledge	33.50 ± 6.36
Attitude	51.37 ± 5.33
Possibility	21.34 ± 3.60
Willingness	34.41 ± 4.34
Use	32.03 ± 4.16
Total	176.66 ± 14.74

**Table 2:** The average score of evidence-based practice based on demographic variables.

Variable		Mean ± SD	*P* value
Gender	Male	172.24 ± 14.10	0.740
	Female	173.05 ± 15.13	

Marital status	Single	173.02 ± 14.25	0.039
	Married	164.61 ± 19.72	

Prior familiarity with EBP	Yes	179.84 ± 15.02	0.002
	No	170.68 ± 14.10	

Interest in the field of study	Positive	174.28 ± 13.64	0.001
	Negative	161.68 ± 18.64	

Research experience	Yes	179.54 ± 15.34	0.001
	No	169.94 ± 13.63	

Willingness to participate in Master's entrance exam	Yes	176.76 ± 13.27	0.019
	No	168.22 ± 15.06	

**Table 3:** Correlation between the average of the EBP score and the age and GPA of the students.

Variable	EBP score
Age	*R* = −0.075
	*P*=0.367

GPA	*R* = 0.369
	*P*=0.001

## Data Availability

The data used to support the findings of this study are available from the corresponding author upon request.
